# Parallel Hough Transform-Based Straight Line Detection and Its FPGA Implementation in Embedded Vision

**DOI:** 10.3390/s130709223

**Published:** 2013-07-17

**Authors:** Xiaofeng Lu, Li Song, Sumin Shen, Kang He, Songyu Yu, Nam Ling

**Affiliations:** 1 Shanghai Key Laboratory of Digital Media Processing and Transmissions, Shanghai Jiao Tong University, Shanghai 200240, China; E-Mails: luxiaofeng@shu.edu.cn (X.L.); syyu@sjtu.edu.cn (S.Y.); 2 School of Communication and Information Engineering, Shanghai University, Shanghai 200072, China; E-Mails: leo_shu@126.com (S.S.); hkzy2001@gmail.com (K.H.); 3 Department of Computer Engineering, Santa Clara University, Santa Clara, CA 95053-0566, USA; E-Mail: nling@scu.edu

**Keywords:** straight line detection, parallel Hough Transform, Canny edge detection, FPGA, embedded vision

## Abstract

Hough Transform has been widely used for straight line detection in low-definition and still images, but it suffers from execution time and resource requirements. Field Programmable Gate Arrays (FPGA) provide a competitive alternative for hardware acceleration to reap tremendous computing performance. In this paper, we propose a novel parallel Hough Transform (PHT) and FPGA architecture-associated framework for real-time straight line detection in high-definition videos. A resource-optimized Canny edge detection method with enhanced non-maximum suppression conditions is presented to suppress most possible false edges and obtain more accurate candidate edge pixels for subsequent accelerated computation. Then, a novel PHT algorithm exploiting spatial angle-level parallelism is proposed to upgrade computational accuracy by improving the minimum computational step. Moreover, the FPGA based multi-level pipelined PHT architecture optimized by spatial parallelism ensures real-time computation for 1,024 × 768 resolution videos without any off-chip memory consumption. This framework is evaluated on ALTERA DE2-115 FPGA evaluation platform at a maximum frequency of 200 MHz, and it can calculate straight line parameters in 15.59 ms on the average for one frame. Qualitative and quantitative evaluation results have validated the system performance regarding data throughput, memory bandwidth, resource, speed and robustness.

## 1. Introduction

Optimal straight line detection is a considerable step for several embedded vision applications, and now the largest research focus is based on the Hough Transform (HT) [1]. The straight line detection has been widely used in many industrial applications like image analysis, smart robots, intelligent vehicles, and pattern recognitions [2]. Generally, the least squares method (LSM) can easily obtain the slope and intercept of a straight line through a scanning of the edge image, but this method is very sensitive to noise [3,4]. HT is widely used for its accuracy and robustness in digital low-definition and still images [5], although it is memory and computation demanding.

Current trends in complex computing architectures integrate Field Programmable Gate Arrays (FPGAs) as a competitive alternative due to its parallelism to accelerate computational performance for embedded vision applications. There are many FPGA implementation research areas, such as lane detection [6], stereo vision processing [7,8], high-speed face detection [9], image segmentation [10], background subtraction [11], and more. These interesting real-time applications demonstrate the great computational performance of the FPGA architecture. In this paper, a novel real-time FPGA architecture based on a modified Canny edge detection method and spatial angle-level optimized parallel Hough Transform (PHT) algorithm to accomplish straight line detection in high-definition video sequence is proposed.

In this proposed real-time straight line detection system, detection of edge pixels is a basic task that has a significant influence on the performance of follow-up calculation. Currently, edge detection algorithm mainly includes the following two approaches: (1) Classical gradient differential operator, such as Sobel operator, Canny operator [12], Prewitt operator, and Roberts operator; (2) Laplacian algorithms [13], like Gaussian, Marrs Hildreth, and more. As we know, the edge is determined by the brightness difference of one pixel to the others, and we can identify the edge pixels if the brightness changes sharply [14]. Generally, the Canny operator proposed by Canny in 1986 is used in various edge detection applications, and it can obtain single pixel edge feature images. However, this method suffers from false edges or missing edge information in complex backgrounds; in addition, resource requirement and hysteresis thresholds scheme make extensive processing worse. Troubled by these factors, hardware acceleration architecture on FPGA to Canny operator becomes an attractive alternative [15]. In our earlier work [16], an improved high-speed Canny edge detection architecture based on FPGA was proposed. In that implementation, the gradient is calculated by the second harmonic of the variable parameters to simplify complex arithmetic into basic logic operations. On the basis of this modified edge detection algorithm, we also proposed a circle detection architecture utilizing optimal parameter statistics model in FPGA as a vision application [17].

The classical HT converts the edge feature image into a new domain called the Hough parameter space as a popular method for straight line detection. We can obtain the computational result in Hough space by mapping the parameter points to image space. Each point in Hough space corresponds to a line in the initial image. Reasonable use of peak information in the Hough space is the common denominator of all HT-related detection methods [18]. A large amount of calculation parameters need to be stored and this seriously constrains the HT performance of accuracy and robustness [19]. Simultaneously, HT research has been widely applied to detect a variety of shapes in images, such as straight lines [5], elliptical objects [20], arbitrary objects [21], rectangles [22], triangles [23], and circles [24].

Duda *et al.* [5] were the first to propose the parameter space transform method applied to straight line detection in digital images. This transform converts the Cartesian coordinates to parameter coordinates (*ρ*,*θ*) for line equations. In the Hough parameter space, the corresponding line equations intersect at (*ρ*,*θ*). Through accumulation of the intersection points, we can find a specific parameter (*ρ*,*θ*) with a peak value that respects to the candidate straight line [25]. But this method suffers from great execution time and resource requirement. To overcome this problem, there are many modifications of acceleration and accuracy improvement in the software algorithm of HT. Rau *et al.* [26] utilized the principal axis analysis method to speed-up the straight line parameter estimation. Duquenoy *et al.* [27] used spatial under-sampling and anticipated maxima detection to accelerate the algorithm execution. Li *et al.* [28] and Illingworth *et al.* [29] proposed coarse-to-fine techniques to improve straight line parameter calculation accuracy.

Furthermore, there are acceleration solutions based on different hardware architectures to increase processing speed for tremendous real-time performance and avoid high development costs. Examples include graphics processing unit (GPU) [30,31], digital signal processor (DSP) [32], coordinate rotation digital computer (CORDIC) [33,34], distributed arithmetic (DA) architecture [35], array processor (AP) [36], and FPGA architecture [2,37-39]. However, the calculation speed and hardware resource on GPU or DSP cannot be satisfied to the requirement of computer vision systems. Especially, for the performance and energy comparison on image processing in [31], FPGA can implement the acceleration of up to 11 × and 57× compared to that of GPU and multi-cores, respectively. FPGA is the most energy-efficient architecture from the experiments; with one and two orders of magnitude lower energy consumption than that of the same implementations in GPU and CPU, respectively. Zhou *et al.* [33] proposed the use of CORDIC algorithm for fast HT, and Karabernou *et al.* [34] used the gradient and CORDIC for real-time straight line detection architecture. But CORDIC and DA architecture both require many iterations for accurate parameters calculation in HT space and the number of iterations has a significant impact on computational complexity, resource requirement, and data throughput. The AP architecture is an angle-level parallel algorithm, but its edge feature scanning mode limits its data throughput. Ahmed *et al.* [39] proposed a memory efficient FPGA implementation of HT for line and circle detection, and its HT space was sampled in an efficient way to reduce the size of HT space and memory requirement. However, the sampling rate affected the detection accuracy greatly, and it led to about 50% lower than that of the region-based method. Additionally, Kim *et al.* [2] presented a finite line detection system based on partitioned parallel HT units in Hough space to increase speed and accuracy. However, it was just fit for still image processing based on caching whole image data, and the computational frame rate was improved by increasing the line identification clock frequency. A resource-efficient architecture and implementation of HT on FPGA was presented in [40]. In this processing element (PE) based architecture, the pre-calculated edge feature image was divided into blocks to facilitate algorithm parallelism, and the incrementing property was applied to reduce resource requirement. The major disadvantages of this pixel-level optimized method were that it should cache the entire edge feature image for zero-run-length encoding, and it just implemented the voting process of HT algorithm in resolution 512 × 512 without qualitative and quantitative experimental results. Shang *et al.* [41] roughly prejudged the lines in original image through the directional filter and calculated the exact value later. This method successfully cut back the computation load by narrowing the range of accuracy. In [42], He *et al.* proposed a new algorithm using shift and addition operations to replace the complex trigonometric functions and multiplications in traditional HT, and a new threshold method based on the peak information extraction was presented to eliminate the fake lines. But in this method and implementation, the minimum step of *θ* (Δ*θ*) equaled to 1.79°, and the detection accuracy was limited.

Some attempts in literatures as mentioned above have been made to detect straight lines in low-definition and still images for some vision applications. Many researchers depend on increasing the signal processing frequency for the pre-stored edge image to upgrade computational speed. From the performance and resource consumption perspective, few refine them by the associated optimization of HT software algorithm and hardware architecture applied to real-time high-definition video sequences. So in this paper, we extend the work in [5] and [42], and propose a novel dedicated PHT algorithm and FPGA hardware architecture associated parallel framework for real-time straight line detection for high-definition video sequences. This complete embedded vision system includes high-definition video capture, modified edge feature image calculation, straight line parameter computation, and detection result display. The main contributions of this new framework include the following. First, a new resource-optimized Canny edge detection method is presented to suppress most possible false edges and obtain fewer, but more accurate, candidate edge pixels for subsequent accelerated computation with more enhanced non-maximum suppression conditions, compared with our previous work [16]. Second, instead of acting towards increasing calculation frequency, the novel spatial angle-level PHT algorithm was derived for the 1,024 × 768 resolution real-time video frames to improve the minimum computational step to 0.8952° for the balance of computing speed and parameter detecting accuracy. Finally, optimized with the spatial angle-level parallelism, the novel multi-level pipelined PHT hardware accelerating architecture is proposed for straight line parameter fast estimation and we also present the FPGA implementation in detail. In particular, parallel pipeline calculation units based maximum searching scheme in HT space is described for candidate straight line parameter decision. The proposed framework is mapped on to ALTERA Cyclone IV EP4CE115F29 FPGA with the maximum frequency 200 MHz. Under the guidance of associated optimization both on algorithm and on architecture, this real-time and robust PHT can achieve straight line detection in videos of 1,024 × 768 resolution with a pixel clock of 43 MHz effectively. In addition, this implementation avoids high development costs and obtains enough flexibility, which enables many potential pattern recognition applications.

## 2. Overview of the Proposed Embedded Straight Line Detection Vision System

**Figure 1.**
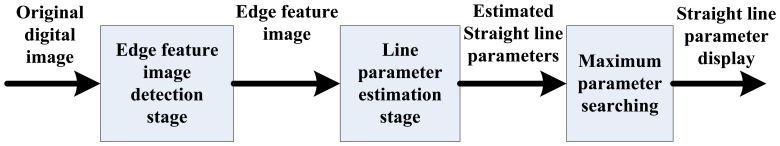
PHT straight line detection algorithm flow.

We primarily present a novel PHT-based straight line detection algorithm and the corresponding architecture implementation on FPGA to deal with the acceleration and accuracy problems of classical HT. As Figure 1 shows, there are two main stages in this algorithm: edge feature image detection stage and straight line parameter estimation stage. In order to obtain the edge feature from original digital image for accelerating computation, a resource-optimized Canny edge detection algorithm is adopted. In the procedure of straight line parameter estimation, a spatial angle-level PHT algorithm derivation for the 1,024 × 768 resolution real-time video sequences is firstly proposed. Then, the associated optimized multi-level pipelined PHT architecture is presented for parameter estimation and implements on a single FPGA. Finally, the maximum searching scheme in HT parameter space for candidate straight line parameter decision is used based on parallel pipeline calculation units.

**Figure 2.**
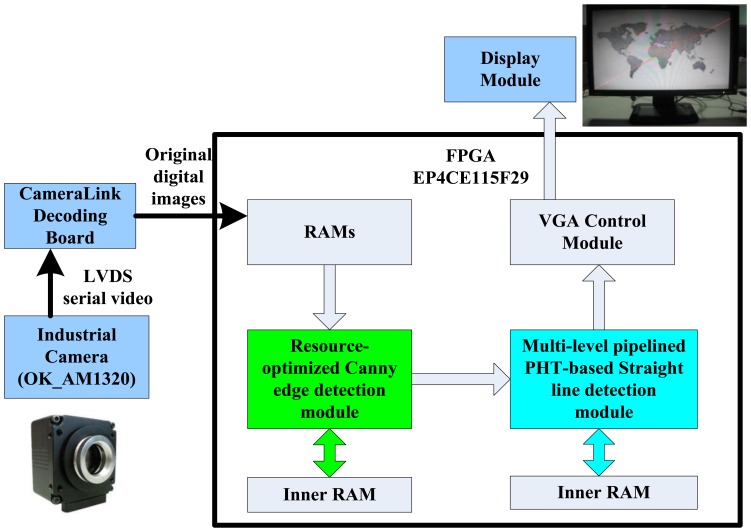
The block diagram of embedded straight line detection system.

The block diagram of the proposed straight line detection architecture is depicted in Figure 2. The ALTERA DE2-115 FPGA evaluation platform with the chip EP4CE115F29 is employed for algorithm implementation. Initially, the industrial camera (OK_AM1320) captures the CameraLink serial video sequence with the 1,300 × 1,024 resolution at 20 frames per second, and through the CameraLink decoding board, the input original RGB images are captured into on-chip RAMs for data processing. The inner RAMs in FPGA are both needed in the edge feature detection module and straight line parameter computation module. The real processed resolution in FPGA is 1,024 × 768 cropped from original image, and calculated straight line parameters are displayed on LCD at a 1,024 × 768 resolution through the VGA control module.

## 3. Resource-Optimized Canny Edge Detection Method

The resource-optimized edge detection algorithm is an extension of our previous work [16]. Here we put forward more enhanced non-maximum suppression conditions compared with [16] to suppress most of possible false edges and obtain fewer, but more accurate candidate edge pixels for subsequent accelerated computation. Of course, some real edge pixels will be lost in this treatment. But in our straight line detection case, the limited loss of some real edge pixels on a candidate line does not have a big impact on the final calculating result, but it can reduce the computational complexity of follow-up line parameter estimation. The experimental results also prove the validity of this approach.

**Figure 3.**
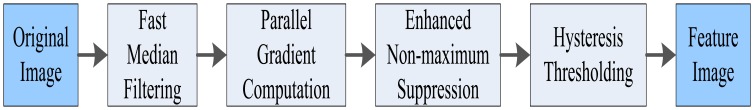
Flow chart of resource-optimized canny edge detection algorithm.

In the classical Canny algorithm, it is difficult to detect accurate edge features in complex backgrounds, because the smoothness of Gaussian filtering with artificial parameters can lead to excessive smoothing (losing edge information) or insufficient smoothing (unable to remove noise). Moreover, insufficient non-maximum suppression conditions result in false edges. In this proposed method, parallel and fast median filter was selected to suppress grain noise, and we do not need to set parameters for implementation in FPGA with flexibility [43]. Furthermore, enhanced non-maximum suppression is employed to suppress false edges by presenting more stringent suppression conditions. The modified Canny edge detection flow chart is shown in Figure 3. Accordingly, many parallel computing mechanisms about the fast median filtering, parallel gradient computation, and enhanced non-maximum suppression are explained in detail in the following paragraphs for resource optimization and accuracy improvement.

### 3.1. Fast Median Filtering

**Figure 4.**
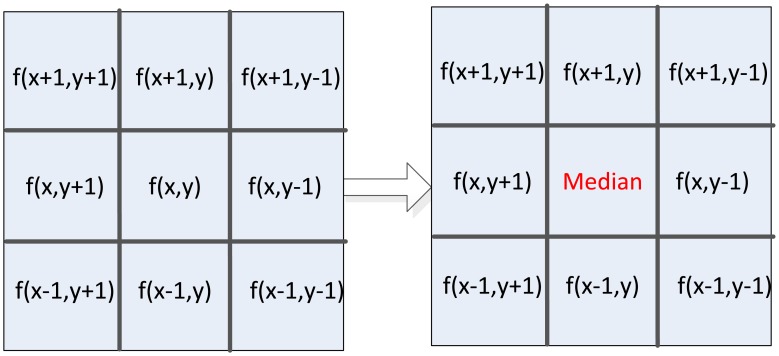
Median filtering diagram.

**Figure 5.**
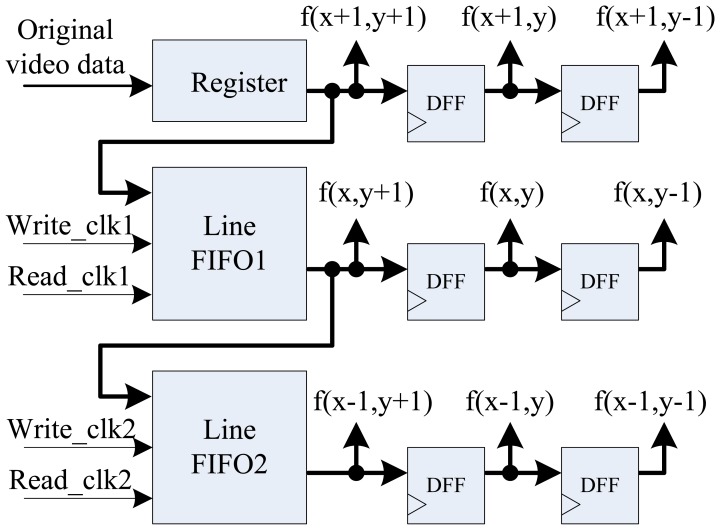
Architecture of a 3 × 3 pixel template cache.

In this new edge detection method, digital image is first smoothened by median filter to reduce noise in the image. In this paper, the 3 × 3 template is used to achieve parallel fast median filtering, and the diagram is shown in Figure 4. Median filtering is used to sort pixel data in the template from small to large, and taking the middle data as the result of current pixel. Usually, median filtering implementation on FPGA needs to cache three image lines and three line FIFOs are required. But in this architecture, we use the pipelined structure by two FIFOs, one register, and six D-type flip-flops (DFF) with delay operations to get the 3 × 3 pixel template. Figure 5 shows the optimal parallel hardware architecture of template pixel cache for computation.

**Figure 6.**
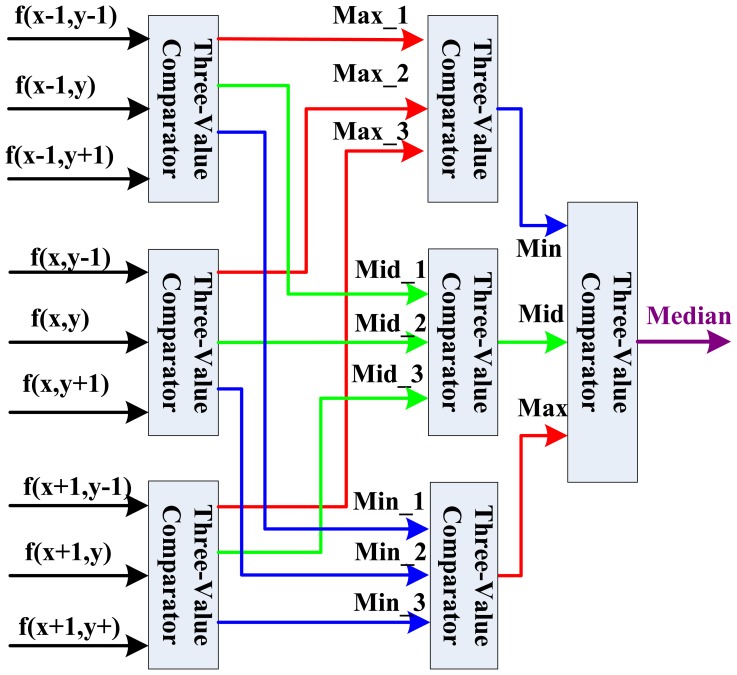
Calculation architecture of parallel fast median filtering.

After the nine template pixel data is obtained, we design a new parallel sorting and comparing architecture based on the parallelism of FPGA as shown in Figure 6 for real-time computation. In this parallel calculation method, seven three-value comparators are needed to build a three-level computing architecture.

In the first level, we divide the nine values into three groups, and three three-value comparators can calculate in parallel with a two clock delay in this level to decide the maximal value (Max), middle value (Mid), and minimal value (Min). For the second level of this architecture, three maximal values, three middle values and three minimal values are assigned into respective groups. Through data analysis, it is clear that in the group of three maximal values, just the minimal value will be the final candidate middle data in nine pixels. So in this group, the Min is selected for the third level middle data comparison. In the middle values group in the second level, just the middle value will be the final candidate middle data. Similarly, in the minimal values group in the second level, just the maximal value will be the final candidate middle data. Finally, in the third level comparison, the real middle data can be decided by a three-value comparator. Therefore, the fast median filtering can be done after a six clock delay.

### 3.2. Parallel Gradient Computation

Equations (1) and (2) show the 3 × 3 pixel template based horizontal gradient *G_x_* and vertical gradient *G_y_* calculation. Then the total gradient *G*(*x*, *y*) can be calculated as in Equation (3) with a sum of two gradients' absolute values. In FPGA implementation, the *N*-th power operations of 2 can be converted to the fast *N*-bit right shifts. And the selection of *N* can be made empirically through experiments:

(1) $$G_{x}=\left[f\left(x+1,y\right)-f\left(x-1,y\right)\right]/2^{N}$$

(2) $$G_{y}=\left[f\left(x,y+1\right)-f\left(x,y-1\right)\right]/2^{N}$$

(3) $$G\left(x,y\right)=\left|G_{x}\right|+\left|G_{y}\right|$$

**Figure 7.**
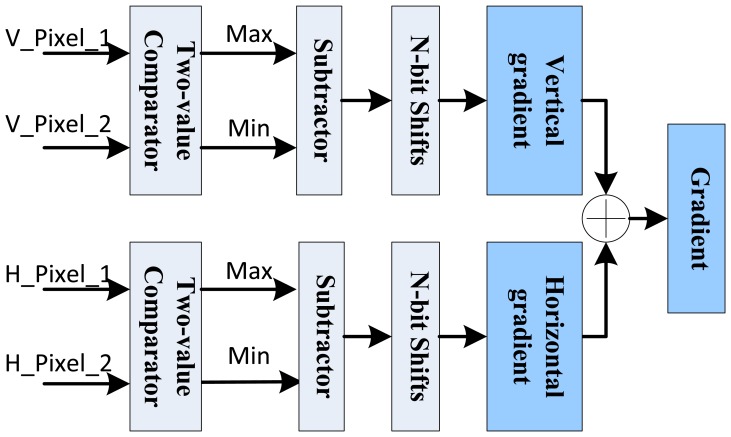
Parallel gradient computation architecture.

In order to reduce the computational complexity in parameter coordinates, it is also needed to record whether the gradient is positive or negative as well as its absolute value. As Figure 7 shows, two two-value comparators are used to decide the actual differences of pixel values. Based on this difference, the gradient absolute value is calculated with corresponding subtractor. Finally, through the parallel comparators and basic logic operations, the gradient can be obtained easily and fast.

### 3.3. Enhanced Non-Maximum Suppression

As expressed in Equation (4), inverse trigonometric function calculation is needed in the classical Canny algorithm to determine the gradient direction in non-maximum suppression, but inverse trigonometric calculation is complex and resource consumption in FPGA architecture:

(4) $$\lambda =\arctan\left(G_{y}/G_{x}\right)$$

**Figure 8.**
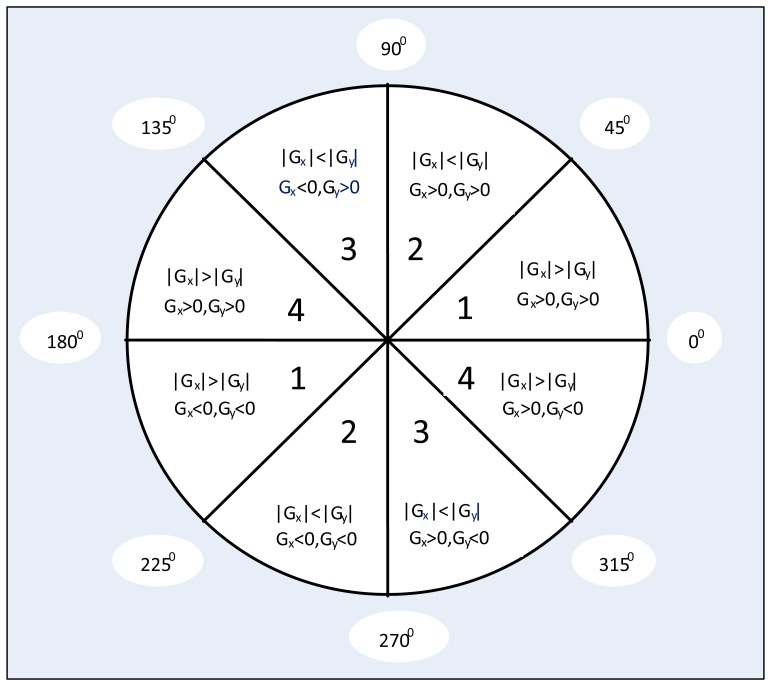
Eight gradient directions division diagram.

In this proposed algorithm, we divide the gradient directions into eight regions as Figure 8 shows, and every direction region contains a range of a 45 degree angle. Depending on whether the gradient is positive or negative and its absolute value difference in the horizontal and vertical directions, we can roughly find out the gradient direction of current pixel. This gradient direction definition can greatly reduce computational region and FPGA resource requirement. Moreover, through the gradient direction standardization and all of the non-peak amplitudes suppression, we can refine the object edge in digital image.

For example, if gradient direction of one pixel is at the range of 0° to 45° or 180° to 225°, the gradient value of this pixel along these two directions can be measured by *M*(*a*) and *M*(*b*) as Equations (5) and (6) shown. In the classical Canny algorithm, if conditions *G*(*x*, *y*)>*M*(*a*) and *G*(*x*, *y*)>*M*(*b*) are both met at the same time, we treat this pixel with the gradient *G*(*x*, *y*) as an edge feature point. In this proposed method, through experimental empiricism, we further add four enhanced suppression conditions defined as Equation (7) to Equation (10) to suppress the potential false edges effectively. These conditions mean that the gradient of the candidate pixel must be larger than that in the neighborhood four directions. The cost of doing under these conditions is losing some of the true edge pixels, but it has no significant effect on the final calculation results for specific length straight line parameter estimation, and the computational complexity will be reduced for a more accurate feature edge, as experimental results demonstrated. For the pixels in other directions, corresponding operations and judgments can be used similarly:

(5) M(a)=[G(x+1,y−1)+G(x,y−1)]/2

(6) M(b)=[G(x,y+1)+G(x−1,y+1)]/2

(7) G(x,y)>G(x+1,y−1)

(8) G(x,y)>G(x,y−1)

(9) G(x,y)>G(x,y+1)

(10) G(x,y)>G(x−1,y+1)

### 3.4. Dual-Threshold Detection

Finally, our dual-threshold detection implementation is based on the verification mechanism proposed by Mondal *et al.* [44]. The higher threshold value (HTV) and the lower threshold value (LTV) are selected to track the remaining candidate pixels that have not been suppressed. Verifications were implemented against the candidate pixels by selected HTV and LTV, respectively. The candidate pixel is then chosen as an edge feature pixel, one situation is that its gradient value is greater than HTV, and another situation is that its gradient value is between the LTV and HTV, but it is connected to an edge pixel directly. In the other cases, the pixel will be declared as a non-edge pixel. Both of LTV and HTV can be calculated empirically by experiments. In our design, HTV is set to 90, and LTV is set to 20 to obtain continuous and detailed edges.

## 4. Multi-Level Pipelined PHT Based Straight Line Parameter Estimation and Decision

### 4.1. Overview of HT

**Figure 9.**
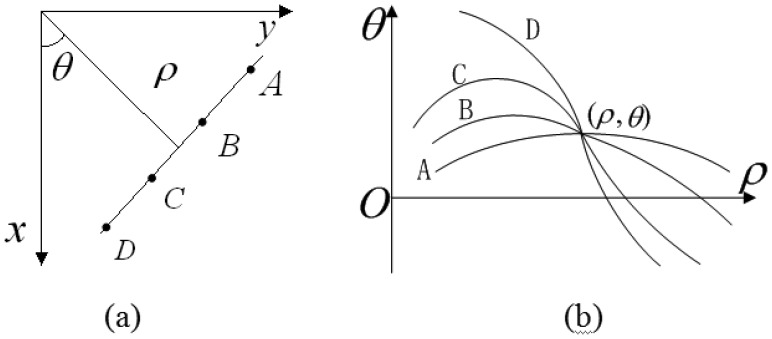
HT representation (**a**) cartesian coordinate; (**b**) parameter coordinate.

HT is a well-known and effective method for straight line detection in digital images. This transform converts the binary edge feature image from Cartesian coordinates (*x*, *y*) into parameter coordinates (*ρ*,*θ*). Equation (11) is used to define a straight line in HT parameter space by parameters *ρ* and *θ*. According to the coordinate correspondence, each point on the straight line in the Cartesian coordinate corresponds to a curve in the parameter space. Meanwhile, all the points on same straight line must correspond to a curve cluster intersected at same point (*ρ*,*θ*) in the parameter space as Figure 9 shows.

Through accumulating the intersection points, we can find the specific parameter (*ρ*,*θ*) with peak information with respect to the candidate straight line. It is clear that trigonometric function and multiplication are the main calculations in the HT expression, and the computational complexity is depending on the number of edge feature pixels and the precise definition of angles:

(11) ρ=xcosθ+ycosθ

### 4.2. Spatial Angle-Level Parallel PHT Algorithm Derivation

Most HT implementations in FPGA platform depend on increasing the processing frequency for low-definition and still images pre-stored in off-chip memory. The sin *θ* and cos *θ* calculated values are stored in inner ROM LUTs. For every edge point, it is needed to traverse the whole LUTs to calculate specific *ρ*, and accumulate to the corresponding counter. Whereas in our vision system, the captured video resolution is 1,300 × 1,024, so the range of *ρ* is (-804, 804), and the range of *θ* is (−*π*/2, *π*/2) as Equation (12) shows. In order to realize real-time computation, it is not allowed to pre-store any edge image information. In this calculation manner, if the minimal step of *θ* is 1°, there are 360 times of the multiplication operation and 180 times of the addition operation, just for one edge point. This calculation style is resource and time consuming:

(12) $$\begin{array}{cc} \rho =x\cos\theta +y\cos\theta & - \end{array}\frac{\pi }{2}\leq \theta \leq \frac{\pi }{2}$$

In this paper, refering to [42], we derivate spatial angle-level optimized parallel HT calculation form for real-time and high-definition video sequence demands. Instead of acting towards increasing calculating frequency that causes great power consumption, this new PHT algorithm adapts to the FPGA hardware architecture as an associated optimization. Considering the trigonometric conversion relations, Equation (12) can be divided into two related parts, as Equation (13) shows. Where *ρ_a_* and *ρ_b_* represent the value of *ρ* when *θ* ∈ [0, *π*/2] and *θ* ∈ [−*π* /2, 0], respectively:

(13) $$\{\begin{array}{l} \begin{array}{cc} \rho_{a}=x\cos\theta +y\sin\theta & 0\leq \theta \leq \frac{\pi }{2} \end{array}\\ \begin{array}{ll} \rho_{b}=x\cos\theta +y\sin\theta & -\frac{\pi }{2}\leq \theta \leq 0 \end{array} \end{array}$$

Now, we further define *θ* = *θ ′* −*π*/2, then the part of *θ* ∈ [−*π*/2, 0] can be expressed as:

(14) $$\begin{array}{cc} \rho_{b}=x\cos(\theta^{\prime }-\frac{\pi }{2})+y\sin(\theta^{\prime }-\frac{\pi }{2})=x\sin\theta^{\prime }-y\cos\theta^{\prime } & 0\leq \theta^{\prime }\leq \end{array}\frac{\pi }{2}$$

After the definition as Equation (14), the two parts of *ρ* can be modified into the same angle range in Hough parameter space as shown in Equation (15). *ρ* can be calculated in parallel in two opposite parts and the calculated angle range of *θ* is cut to half:

(15) $$\{\begin{array}{c} \begin{array}{cc} \rho_{a}=x\cos\theta +y\sin\theta & 0\leq \theta \leq \frac{\pi }{2} \end{array}\\ \begin{array}{cc} \rho_{b}=x\sin\theta^{\prime }+y\cos\theta^{\prime } & 0\leq \theta^{\prime }\leq \end{array}\frac{\pi }{2} \end{array}$$

On the assumption that the minimal step of *θ* is Δ*θ*, and the location of edge point is (*x*, *y*). In Equation (15), the value of *θ* is digitized to 0,Δ*θ*,2Δ*θ*,…,*i*Δ*θ*,…,*π*/2. We then derive the equation in accordance with the discrete values.

When *θ* = 0, Equation (15) can be expressed as:

(16) $$\{\begin{array}{l} \rho_{a0}=x\\ -\rho_{b0}=y \end{array}$$

Next, when *θ* = Δ*θ*, Equation (15) can be expressed as:

(17) $$\{\begin{array}{l} \rho_{a1}=x\cos\Delta \theta +y\sin\Delta \theta =\cos\Delta \theta (\rho_{a0}-\rho_{b0}tg\Delta \theta )\\ -\rho_{b1}=x\sin\Delta \theta -y\cos\Delta \theta =\cos\Delta \theta (-\rho_{b0}-\rho_{a0}tg\Delta \theta ) \end{array}$$

and so on; when *θ* = (*i* + 1) Δ*θ*, the new PHT calculation can be indicated as follows:

(18) $$\{\begin{array}{l} \rho_{a(i+1)}=\cos\Delta \theta (\rho_{ai}-\rho_{bi}tg\Delta \theta )\\ -\rho_{b(i+1)}=\cos\Delta \theta (-\rho_{bi}-\rho_{ai}tg\Delta \theta ) \end{array}$$

Obviously, if we traverse all the possible discrete values of *θ*, the current calculation result is related to the last one. With further approximation, if *tg*Δ*θ* = 1/2^6^, then Δ*θ* = 0.8952° and cosΔ*θ* = 0.9999 ≈ 1, so Equation (18) can be further approximated to Equation (19). This approximate expression can be calculated in parallel and just include basic addition and shift operations:

(19) $$\{\begin{array}{l} \rho_{a(i+1)}=\rho_{ai}-\rho_{bi}*\frac{1}{2^{6}}\\ -\rho_{b(i+1)}=-\rho_{bi}-\rho_{ai}*\frac{1}{2^{6}} \end{array}$$

### 4.3. Multi-Level Pipelined PHT Architecture Based Straight Line Parameter Estimation

**Figure 10.**
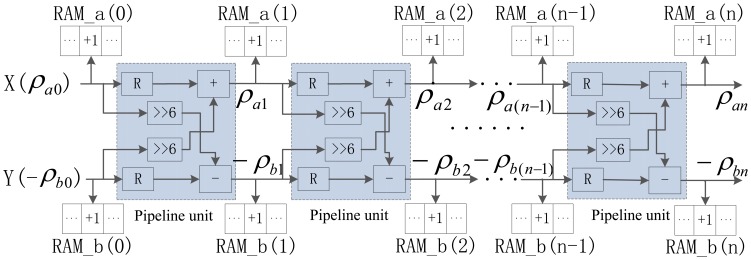
Multi-level pipelined PHT architecture.

Through Equation (19), we successfully convert traditional multiplication and trigonometric function operations in HT algorithm into addition and shift operations, which is adaptive to the parallel calculation structure in FPGA circuit. If Δ*θ* = 0.8952°, the steps of *θ* is equal to 101. For each edge pixel, our parallel HT needs to do 101 times the calculation in the range of *θ* ∈ [0, *π*/2] for straight line parameter estimation, which greatly improves calculation accuracy. Meanwhile, the improvement of calculation accuracy brings substantial computational load increase and more effective computing structure is needed. According to the relationship between two consecutive calculations in Equation (19), associated with spatial angle-level parallelism, a novel multi-level pipelined PHT calculation architecture is proposed as in Figure 10, to improve computational efficiency.

In this multi-level pipelined PHT architecture, the straight line parameters can be calculated through 101 pipeline units (*n* = 0,1,…,100) for one edge pixel, and every pipeline unit represents an angle stepper calculation. All of these calculation units are subject to pipelined computing, and the output of any unit is served as input parameter to the next pipeline unit. The advantage of thus pipeline structure is that the calculation of 101 pairs of (*ρ*,*θ*) for one edge pixel can be completed in just one clock cycle.

For each pipeline unit, the hardware architecture in FPGA needs two registers, two 6-bit shifts, one adder, and one subtractor. The initial inputs *X*(*ρ_a0_*) and *Y*(−*ρ_b0_*) of this pipelined calculation architecture are the location of the corresponding edge pixel. Each unit has two accumulators RAM_a(i) and RAM_b(i) for parameter accumulating. For the *i-th* calculation unit, the calculated value of *ρ_ai_* is treated as the corresponding memory address in RAM_a(i), and the value in this address plus one. For *ρ_bi_*, a similar operation is taken. In two accumulators, the accumulating address just need the value of *ρ* without *θ*, because to every unit and corresponding accumulator RAM_a(i) and RAM_b(i), the corresponding value of *θ* is identified by i*Δ*θ* or (i*Δ*θ*−*π*/2) previously.

### 4.4. Parallel Pipeline Units Based Maximum Searching Scheme

**Figure 11.**
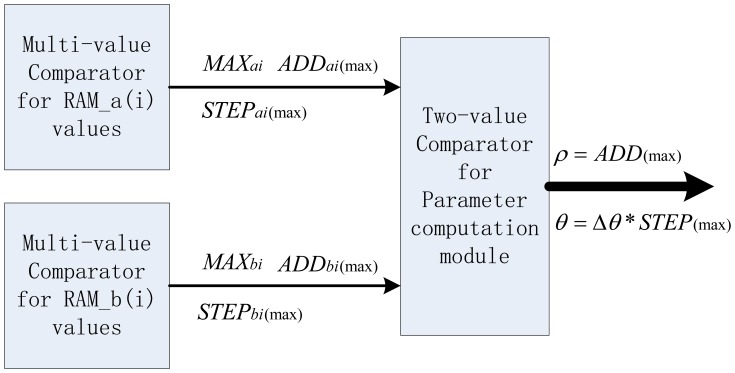
Block diagram of the maximum search scheme for parallel pipeline units.

After parameter estimation for all of the candidate edge pixels in one image with multi-level pipelined PHT architecture, RAM_a(i) and RAM_b(i) will contain all the candidate accumulating results. Current work is to find the peak accumulating information in these two inner RAMs for all of intersection points, and this peak corresponds to the candidate longest straight line. To RAM_a(i) or RAM_b(i), through the multi-value comparators, it is easy to find the maximum for FPGA implementation respectively. The maximum search results in RAM_a(i) are the three parameters: the accumulating maximum value *MAX_ai_*, the address of the accumulating maximum value *ADD_ai_*_(max)_, and the step number of the accumulating maximum value *STEP_ai_*_(max)_. There is a similar operation to RAM_b(i) to get the corresponding three parameters. In the follow-up straight line parameter computation module, a two-value comparator is used to determine the final maximum between *ADD_ai_*_(max)_ and *ADD_bi_*_(max)_ in both RAM_a(i) and RAM_b(i). Referring to the initial HT definition, we can obtain the specific parameter (*ρ*,*θ*) for this detection procedure, where *ρ* equals the final maximum address *ADD*_(max)_ in RAM_a(i) or RAM_b(i), and *θ* equals the step number of the final maximum *STEP*_(max)_ multiplied by) Δ*θ* if the maximum is in RAM_a(i), or multiplied by) Δ*θ* and subtract *π* /2 if the maximum is in RAM_b(i). The block diagram of parallel pipeline units based maximum search scheme is shown in Figure 11. Obviously, this parallel searching scheme in HT parameter space further improves the real-time performance of this detection architecture.

### 4.5. The Integral Multi-Level Pipelined PHT Based Straight Line Detection Algorithm

**Figure 12.**
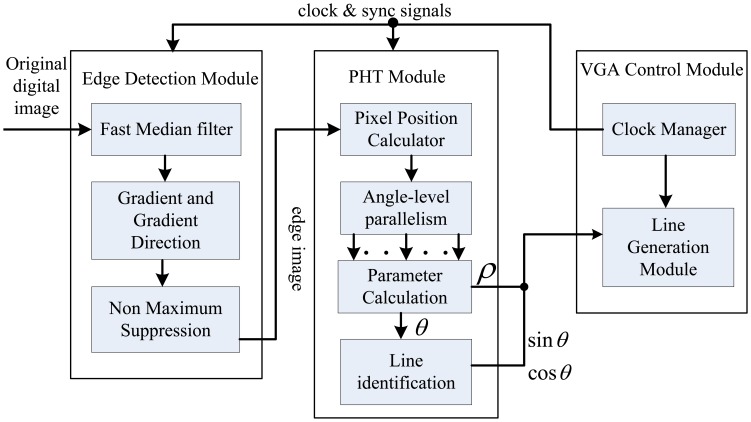
Integral PHT algorithm diagram.

The integral multi-level pipelined PHT based straight line detection algorithm is shown in Figure 12. The arrows indicate the direction of data flow and clock control. We can get the edge image by the resource-optimized edge detection module as described in Section 3. Every candidate edge pixels are computed by multi-level parallel pipeline units and the parameters of real straight line can be determined in parameter calculation circuit in the PHT module. In order to draw a red indicating line on the display image to show the location and direction of detected line, we use the CORDIC processing unit in the FPGA to effectively calculate the value of sin *θ* and cos *θ* for straight line identification.

## 5. Evaluation

In this section, we propose this embedded vision system based on a FPGA evaluation platform. We evaluate the performance of the FPGA-based straight line detection with throughput, maximum error, memory access bandwidth, and computational time. In addition, we also present qualitative and quantitative experimental results for the accuracy and robustness of our proposed algorithm.

### 5.1. Embedded Vision System Based on FPGA

**Figure 13.**
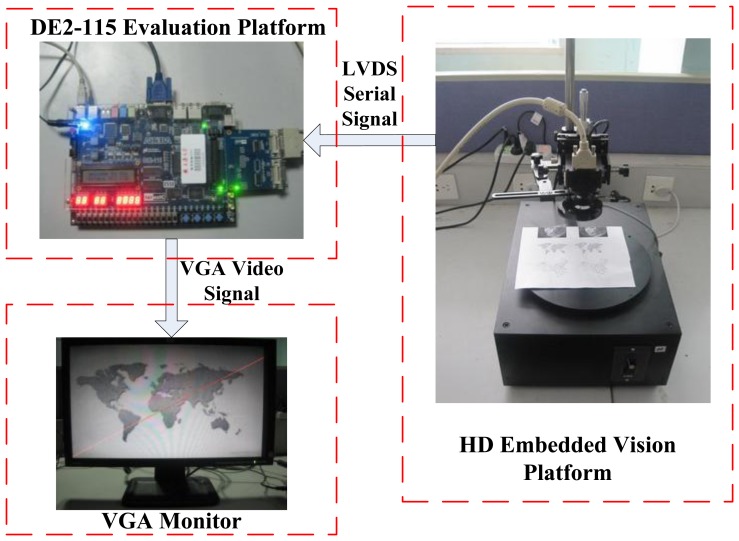
FPGA based embedded straight line detection vision system.

The proposed architecture has been evaluated on ALTERA DE2-115 platform with Cyclone IV EP4CE115F29 FPGA and QuartusII version 10.0 synthesis tool, with the maximum operation frequency of 200 MHz, as Figure 13 shows. This embedded straight line detection vision system can process digital video of 1,024 × 768 resolution cropped from the high-definition LVDS video signal with 1,300 × 1,024 resolution captured by ALTERA DE2-115 evaluation platform through an expansion board CLR_HSMC and CameraLink protocol. This hardware implementation takes 0.24 μs to calculate the edge feature for one pixel and 15.59 ms on the average to compute straight line slope and intercept parameters for one frame, which ensure real-time performance. Finally, the results are displayed with a VGA interface of 1,024 × 768 resolution.

### 5.2. Performance of FPGA Architecture Implementation

#### 5.2.1. Performance Comparison among Different Approaches

In our hardware architecture and implementation, the fraction part *F* is represented in 15 bits. In the definition in Chen *et al.* [40], the maximum error of *ρ* introduced by incremental computation is calculated by (*W* + *H*) × 2^−(*F* +1)^, where *W* is the width and *H* is the height of the processed image. Besides, the throughput per cycle is defined as the average number of computed *ρ* values per cycle, and the throughput is defined as millions of *ρ* values per second. Table 1 presents the performance comparison among different approaches with different processing resolutions.

If the fractional part is 15, the maximum error from Chen *et al.* [40] is 0.012, and the maximum error of our proposed architecture is 0.027. Because our multi-level pipelined architecture can calculate 101 *ρ* values per cycle, the throughput per cycle of our proposed implementation is much higher than the other four approaches. Furthermore, our PHT architecture could achieve great higher throughput of 20,200 compared to other approaches with the cost of a large amount of on-chip memory consumption, although the maximum frequency of our architecture is lower than other approaches. Obviously, enhancing the throughput of hardware architecture is much more important than other requirements in real-time vision applications [40]. In addition, with respect to processing target, still images with low-definition are used in the other four methods whereas our proposed algorithm can deal with highest definition resolution video sequences without any off-chip memory consumption.

#### 5.2.2. Memory and Bandwidth

As a PE-based resource-efficient FPGA architecture, the approach in [40] can greatly reduce the required memory bandwidth and on-chip memory bits, depending on pixel-level optimization. In that method, the voting results are temporarily stored in the on-chip memories and then be transferred to the off-chip memory after completing one angle computation of all the edge feature pixels. In [40], the required memory access bandwidth is calculated by Equation (20), where *K* = 180 is the number of angles, *DataWide* = 9 is the vote memory address width, and the processing image resolution is 512 × 512. In addition, the off-chip memory used in [40] includes the binary feature image store part (2,097,152 bits) and vote memory data transfer store part (1,172,880 bits) of 180 angles at least.

In our proposed multi-level pipelined PHT architecture based on spatial angle-level optimization, after clock delay, it can calculate 101 angles per cycle for one edge feature pixel and get the straight line parameters through peak value search for current pixel. So, in our proposed architecture, the memory address width is 16, and the required memory access bandwidth usage is much larger than [40] as Table 2 shows, because of the higher processing image resolution (1,024 × 768) and higher angle accuracy (Δ*θ* = 0.8952°). Accordingly, our proposed FPGA architecture does not require any off-chip memory to cache image or accumulated results, but this advantage is built on the basis of the consumption of a large number of on-chip memories:

(20) $$\text{Bandwidth}=\sqrt{W^{2}+H^{2}}*K*\text{DataWidth}$$

#### 5.2.3. Calculation Time

Table 3 shows the calculation time comparison between our proposed architecture and other three methods on different platforms.

In Chen *et al.* [40], one binary feature image is pre-stored in the off-chip memory for run-length encoding and PE-based HT computation can reduce the execution time to 2.07–3.61 ms for 512 × 512 image resolution without the edge detection procedure. In our proposed PHT architecture, the processing target is real-time video with 1,024 × 768 processed resolution, and it executes the PHT algorithm on edge pixels sequentially. The execution time of straight line detection in our proposed architecture is 15.59 ms on average, which includes the parameter computation time of 0.02 ms. In Table 3, (a-1) to (a-4) refer to the experimental testing images in Figure 16 and direct HT computation on PC needs more than one second to process an image with MATLAB simulation tools. It is clearly that if taking into account the resolution factor and edge pixel number factor of the processed image, our execution time is similar to [40], and all of the methods implemented on FPGA can ensure real-time processing.

#### 5.2.4. Synthesis Result on Target FPGA

Table 4 shows the synthesis summary of our proposed FPGA architecture resource consumption. From this compiled report, it is clear that the logic element (LE) utilization includes combinational LE with no register (13.72%), combinational LE with a register (10.38%), and sequential LE (1.61%). The total LE used in this implementation is 29,431, and accounts for 25.71%, but it consumes a lot of FPGA inner on-chip memory bits as the parallel pipeline units in PHT. Therefore, 377 M9Ks (True dual-port RAM blocks with 9 K bits of memory) and many block memory bits (3,052,544 bits) are used to construct large amounts of parameter accumulators in pipeline units. Due to numerous variables definition and middle data buffer, the dedicated logic registers utilization is 11.73%. The logic array in FPGA consists of logic array block (LAB), with 10 LEs in each LAB. Every LE is a small logic unit for user logic functions implementation. The usage of embedded multiplier elements is very small, and many multiplications are converted into additions and shift operations because of our angle-level parallel PHT algorithm derivation. In addition, one phase-locked loops (PLL) is used for the high frequency clock generation.

From the work of Fowers *et al.* [31], FPGA can implement the acceleration of up to 11× compared to GPU, and FPGA is the most energy-efficient architecture with one and two orders of magnitude lower energy than the same implementation in GPU and CPU for the same image processing task. Table 5 is the power consumption report of our proposed straight line detection circuit. The total thermal power dissipation is 640.89 mW, and the core dynamic thermal power dissipation accounts for the most part (64.73%). Additionally, the I/O thermal dissipation consumes more power than core static thermal dissipation obviously.

### 5.3. Qualitative Experimental Results for Resource-Optimized Canny Edge Detection

**Figure 14.**
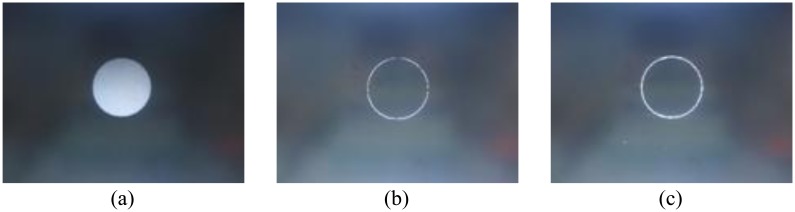
(**a**) Circle image; (**b**) Result of [12]; (**c**) Result of proposed canny.

**Figure 15.**
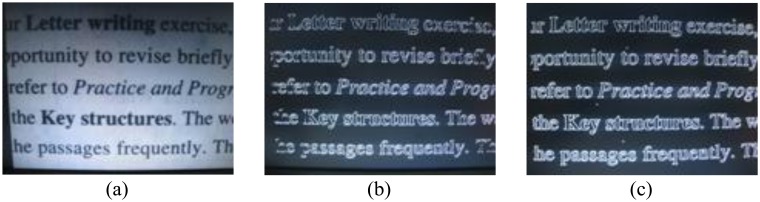
(**a**) Article image; (**b**) Result of [12]; (**c**) Result of proposed canny.

**Figure 16.**
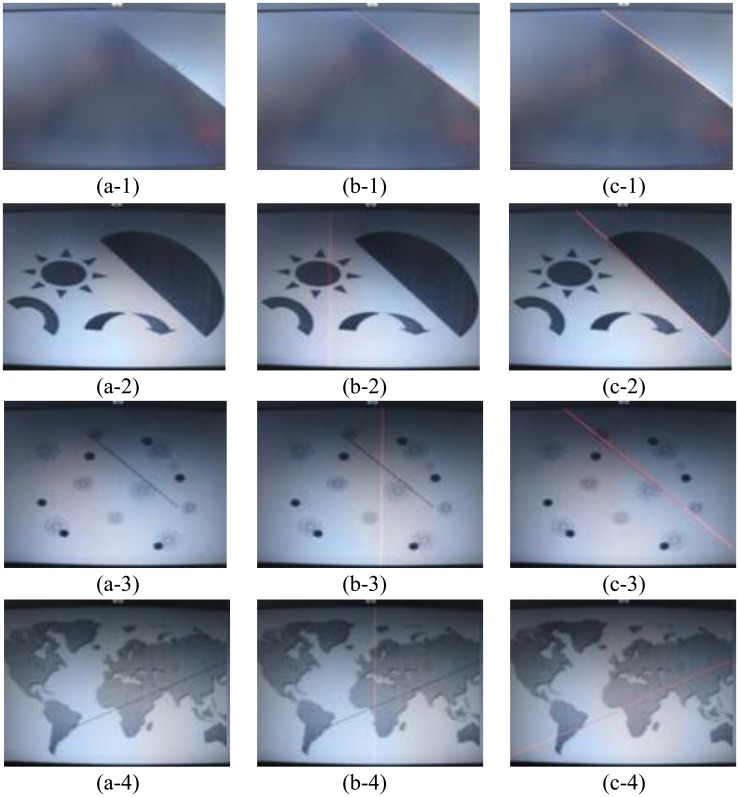
The experimental comparisons of LSM and PHT method: (a-1)–(a-4) are the original images; (b-1)–(b-4) are the results of LSM of Ji *et al.* [3]; (c-1)–(c-4) are the result of the proposed PHT.

Figures 14 and 15 compare the qualitative experimental results between classical Canny [12] and our proposed resource-optimized Canny with enhanced non-maximum suppression conditions. Obviously, the proposed modified Canny edge detection method can obtain more continuous and sharp edges, and the false edges are significantly reduced in some cases. In the circle image detection results, the edge image obtained by classical Canny is discontinuous; and in the article image results, taking the words “Key” and “passages” for example, some edge information obtained by classical Canny is lost, but in modified Canny case, most of the characters and the corresponding edge information are retained. And the tested frames shown in Figures 14, 15 and 16 are generated by ourselves.

### 5.4. Qualitative Experimental Results for Straight Line Detection

Figure 16 shows the qualitative experimental results comparison of LSM [3] and the proposed PHT method on FPGA platform. The first column is the original image, the second column is the result of LSM, and the third column is the result of our proposed PHT. In the first experiment as pictures (a-1) to (c-1), the original image is very simple, so LSM and PHT both can detect the straight line accurately as the red lines show. However, in the second, third, and fourth experiments, the LSM fails to detect the location of straight line in images, but the proposed PHT method obtains the exact straight line object. In these three images, the backgrounds are complex and have many other interference shapes. LSM is very sensitive to the background of testing images, but our proposed PHT method is robust and accurate because of the associated optimization of spatial angle-level PHT software algorithm and multi-level pipelined PHT hardware accelerating architecture.

### 5.5. Quantitative Experimental Results for Straight Line Detection

**Figure 17.**
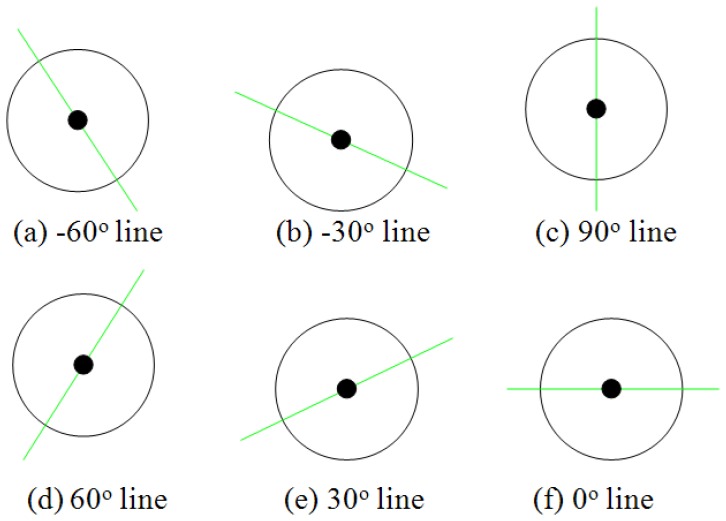
Accuracy and robustness testing samples.

**Figure 18.**
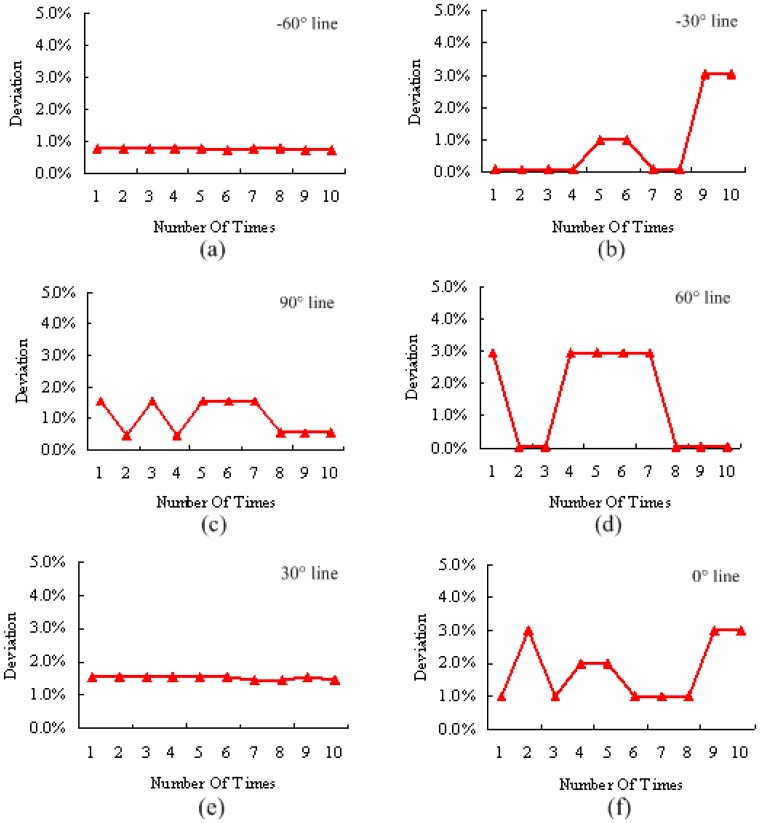
The deviations of tested angles. (**a**) deviation of −60° angle line; (**b**) deviation of −30° angle line; (**c**) deviation of 90° angle line; (**d**) deviation of 60° angle line; (**e**) deviation of 30° angle line; (**f**) deviation of 0° angle line.

From the above qualitative experimental results, our proposed PHT algorithm can detect single straight line in complex background correctly. In this subsection, we present quantitative experimental results to show the accuracy and robustness of this algorithm and hardware architecture. In Figure 17, six hand-generated testing straight lines are given with the angles of −30°, −60°, 0 °, 30°, 60°, and 90°. To every testing sample, 10 times measurements were carried out in the implemented embedded vision system, and the deviations of detected angles are expressed in Figure 18. These curves show that the results of this algorithm are robust. This proposed architecture has considerable stability and satisfies the accuracy requirement of embedded vision applications.

In this experiment, we defined the average testing deviation rate of every angle situation (*ATD_i_*) as in Equation (21) shown, where *N* is the measurement numbers, and *TD_i_* is the tested deviation. The maximum and minimum deviations of every angle situation are defined as *MXTD_i_* and *MNTD_i_*, respectively. In addition, Equation (22) defines the average testing deviation (*ATD*) of all the measured angles and *M* is the testing sorts. Finally, Table 6 shows the quantitative experimental results of straight line detection angle deviation. It is clear that the average testing deviation is 1.236% and this result already has great application value in embedded vision systems:

(21) $${\text{ATD}}_{i}=(\overset{1}{\;}/\underset{N}{\;})\sum_{i=1}^{N}TD_{i}$$

(22) $$\text{ATD}=(\overset{1}{\;}/\underset{M}{\;})\sum_{i=1}^{M}{\text{ATD}}_{i}$$

## 6. Conclusions

In this paper, we have presented a novel PHT algorithm and its FPGA implementation architecture for real-time straight line detection in high-definition video sequences. To obtain fewer but accurate candidate edge pixels, we enhance the non-maximum suppression conditions by a resource-optimized Canny edge detection algorithm. For real-time straight line detection purpose on high-definition video sequences, a novel spatial angle-level PHT algorithm and the corresponding multi-level pipelined PHT hardware architecture are proposed. This gives us an advantage over existing methods which rely on increasing processor frequency.

The proposed algorithm and architecture have been evaluated on the ALTERA DE2-115 evaluation platform with a Cyclone IV EP4CE115F29 FPGA. Quantitative results, including throughput, maximum error, memory access bandwidth, and computational time, on 1,024 × 768 resolution videos are presented and compared with four representative algorithms on different hardware platforms. Due to the PHT software algorithm and its implemented architecture associated optimization, we are not limited just to estimate straight line parameters fast and accurately in high-definition video sequences. This robust and effective embedded vision system has potential applications in various pattern recognition tasks based on high-definition images. Future work consists of exploring spatial and temporal parallelism in the sequence of frames to further reduce computational load.

## Figures and Tables

**Table 1.** Performance comparison among different approaches.

	**Maximum Error of *ρ***	**Throughput per Cycle**	**Maximum Frequency**	**Throughput (M/s)**	**Processing Resolution**
Zhou *et al.* [33]	0.177	2	384 MHz	768	256 × 256
Mayasandra *et al.* [35]	0.125	1/9	500 MHz	56	256 × 256
Chern *et al.* [36]	0.125	1	387 MHz	387	512 × 480
Chen *et al.* [40]	0.012	16	333 MHz	5,328	512 × 512
Proposed method	0.027	101	200 MHz	20,200	1,024 × 768

**Table 2.** Memory and bandwidth comparison results.

	**Processing Image Resolution**	**Off-Chip Memory Bits**	**On-Chip Memory Bits**	**Memory Bandwidth (bits)**
Chen *et al.* [40]	512 × 512	3,270,032	223,360	1,172,880
Proposed	1024 × 768	0	3,052,544	2,674,480

**Table 3.** Calculation time comparison.

**Algorithm and Platform**	**Execution Time**		**Processing Image Resolution**
LSM of Ji *et al.* [3] on FPGA	15.57 ms		1,024 × 768
Chen *et al.* [40] on FPGA	2.07–3.61ms		512 × 512
Proposed Method on FPGA	15.59 ms		1,024 × 768
Direct HT Computation on PC	(a-1)	0.93 s	1,024 × 768
	(a-2)	1.26 s	1,024 × 768
	(a-3)	1.62 s s	1,024 × 768
	(a-4)	1.45	1,024 × 768

**Table 4.** FPGA implementation resource consumption.

**Resource Categories**	**Used**	**Available**	**Utilization**
Combinational LE with no register	15,704	114,480	13.72%
Sequential LE	1,839	114,480	1.61%
Combinational LE with a register	11,888	114,480	10.38%
Dedicated logic registers	13,727	117,053	11.73%
LABs	2,589	7,155	36.18%
M9Ks	377	432	87.27%
Block memory bits	3,052,544	3,981,312	76.67%
Embedded Multiplier 9-bit elements	8	532	1.50%
PLLs	1	4	25.00%

**Table 5.** FPGA implementation power consumption.

**Power Summary**	**Power Consumption**
Total thermal power dissipation	640.89 mW
Core dynamic thermal power dissipation	414.83 mW
Core static thermal power dissipation	105.40 mW
I/O thermal power dissipation	120.66 mW

**Table 6.** Quantitative experimental results for straight line detection angle deviation.

	**–60°**	**–30°**	**90°**	**60°**	**30°**	**0°**
*ATD_i_*	0.753%	0.854%	1.016%	1.492%	1.508%	1.791%
*MXTD_i_*	0.764%	3.056%	1.528%	2.948%	1.530%	2.984%
*MNTD_i_*	0.728%	0.072%	0.461%	0.036%	1.456%	0.995%
*ATD*	1.236%					
